# A Step‐Up Underwater Endoscopic Mucosal Resection Strategy with Rescue Underwater Injection Endoscopic Mucosal Resection for Non‐Pedunculated Colorectal Lesions

**DOI:** 10.1002/deo2.70342

**Published:** 2026-05-06

**Authors:** Akihiro Yoshida, Tsuyoshi Ono, Satoshi Ito, Kenta Watanabe, Toru Okuzono, Katsunori Iijima

**Affiliations:** ^1^ Department of Internal Medicine Omori Municipal Hospital Akita Japan; ^2^ Department of Gastroenterology Sendai Kosei Hospital Miyagi Japan; ^3^ Department of Gastroenterology Akita University Graduate School of Medicine Akita Japan

**Keywords:** colorectal polyps, endoscopic resection, step‐up strategy, underwater endoscopic mucosal resection, underwater injection endoscopic mucosal resection

## Abstract

**Objectives:**

Underwater endoscopic mucosal resection (UEMR) is an established treatment for non‐pedunculated colorectal lesions, but securing an adequate resection margin can be difficult in selected cases due to poor visualization or snare instability. To address these limitations, we used a step‐up strategy with UEMR first and selective conversion to underwater injection EMR (UIEMR) when necessary. This retrospective study evaluated the technical outcomes and safety of this step‐up strategy for non‐pedunculated lesions measuring 10–20 mm.

**Methods:**

This single‐center retrospective study included 56 consecutive 10–20 mm non‐pedunculated colorectal lesions treated with UEMR as the first‐line approach between September 2024 and September 2025. When predefined intra‐procedural criteria for UEMR difficulty were met, the procedure was converted to UIEMR. The primary endpoints were technical success (en bloc and R0 resection rates) and procedure‐related adverse events of the step‐up strategy.

**Results:**

Resection was completed using UEMR alone in 31 lesions, whereas 25 lesions required conversion to UIEMR. En bloc resection was achieved in all lesions, and histologically negative margins were reported in all cases. No delayed bleeding or perforation was observed.

**Conclusion:**

For 10–20 mm non‐pedunculated colorectal lesions, a step‐up strategy with UEMR first and selective conversion to UIEMR was feasible and safe and may be a practical treatment option.

Clinical trial registration: N/A.

## Graphical abstract

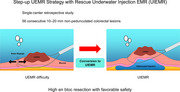

## 1 Introduction

Endoscopic resection of colorectal polyps has been shown to reduce colorectal cancer–related mortality [1], and endoscopic mucosal resection (EMR) is currently established as the standard treatment for non‐pedunculated colorectal lesions [2]. However, as lesion size increases beyond 10 mm, the en bloc resection rate declines, and local recurrence rates after piecemeal resection have been reported to exceed 15% [3, 4]. Incomplete resection contributes to interval colorectal cancers (interval CRCs) [5], with higher rates reported in non‐pedunculated colorectal lesions measuring 10–20 mm [6]. Consequently, achieving complete resection is considered an important quality indicator of endoscopic treatment for colorectal polyps [7].

Underwater EMR (UEMR) has attracted attention as an alternative to conventional EMR. Randomized trials and meta‐analyses have suggested that UEMR improves en bloc and R0 resection rates without increasing adverse events. Nevertheless, the completeness of resection with UEMR remains suboptimal. A randomized controlled trial conducted in Japan reported an R0 resection rate of only 69% for intermediate‐sized (10–20 mm) non‐pedunculated colorectal lesions [8]. Furthermore, a meta‐analysis reported overall R0 and en bloc resection rates of 58% and 70%, respectively [9]. These findings suggest that UEMR alone may be insufficient in some patients.

In clinical practice, UEMR can be technically difficult because of poor visualization or snare slippage. To address these limitations, our institution has introduced underwater injection EMR (UIEMR), which combines submucosal injection with an underwater environment, as a rescue option for UEMR–difficult cases. In this retrospective study, we evaluated whether a step‐up strategy using UEMR first, with conversion to UIEMR when adequate snaring could not be achieved during the procedure, improves outcomes for 10–20 mm non‐pedunculated colorectal lesions.

## 2 Methods

### 2.1 Study Design and Patients

This retrospective single‐center study included consecutive patients who underwent UEMR as the first‐line approach for non‐pedunculated colorectal lesions measuring 10–20 mm between September 2024 and September 2025. Lesions were classified according to the modified Paris classification (0‐Is, 0‐Isp, 0‐IIa, and mixed types), and 0‐Ip lesions were excluded [10, 11]. All procedures were performed by a single endoscopist, a board‐certified endoscopist of the Japan Gastroenterological Endoscopy Society (JGES) with eight years of experience in therapeutic endoscopy. Lesion size was estimated endoscopically using a distal attachment cap and confirmed with an opened snare (15–25 mm). In patients receiving antithrombotic therapy, management followed the JGES guidelines [12]. This study was approved by the institutional review board of our institution (approval number: 3) and conducted in accordance with the Declaration of Helsinki [13]. Written informed consent was waived due to the retrospective design, and an opt‐out approach was used.

### 2.2 Endoscopic Procedures

All procedures were performed using a high‐definition colonoscope (PCF‐H290ZI; Olympus, Tokyo, Japan). A distal attachment cap was used in all cases, and scope insertion was performed under water immersion. After reaching the cecum, mucosal observation was performed under CO_2_ insufflation. Preoperative assessment of invasion depth was primarily performed using white‐light imaging and magnifying narrow‐band imaging. Magnifying chromoendoscopy with pit pattern classification was added when necessary. Lesions showing endoscopic features suggestive of deep submucosal invasion, including markedly irregular vascular patterns corresponding to JNET type 3 or non‐structured pit patterns, were excluded from endoscopic resection. Once the target lesion was identified, resection was initially attempted using UEMR as the first‐line approach. When UEMR was judged to be technically difficult, the procedure was converted to UIEMR. In this study, UEMR difficulty was defined as failure to satisfy any of the following criteria: (1) The snare tip could be stably anchored to the adjacent normal mucosa without repeated slippage; (2) Snaring could be performed with a 1–2 mm margin of surrounding normal mucosa; (3) A circumferential normal mucosal margin could be clearly visualized endoscopically.

UEMR was performed under complete water immersion after luminal deflation using room‐temperature tap water with a water pump system (OFP‐2; Olympus), followed by snaring with surrounding normal mucosa and electrosurgical resection (Video S1). UIEMR was performed when UEMR difficulty was determined according to predefined criteria, under the same underwater conditions with submucosal saline injection, followed by snaring of the elevated mucosa and electrosurgical resection (Video S2). After endoscopic resection, the resection margins were carefully inspected to confirm the absence of residual lesions. For both techniques, polypectomy snares with loop diameters of 15–25 mm and wire diameters of 0.3–0.35 mm were used. A 26‐gauge injection needle was used for submucosal injection. An ESG‐100 electrosurgical generator (Olympus) was used in PulseCut Fast mode with a power setting of 25 W. Lesion resection was performed using the cutting mode only, without coagulation mode. Resection defects were closed using clips.

### 2.3 Histological Examination

Resected specimens were formalin‐fixed and submitted to a specialized pathology center, where histopathological evaluation was performed by pathologists certified by the Japanese Society of Pathology. Histological diagnosis followed the Japanese Classification of Colorectal Carcinoma [14]. Lesions were classified as adenoma with low‐grade dysplasia, adenoma with high‐grade dysplasia, serrated lesions, or carcinoma (pTis/pT1). Histological evaluation was performed blinded to the resection method.

### 2.4 Outcomes

The primary outcomes were the technical success and safety of the overall step‐up strategy, in which UEMR was used first with conversion to UIEMR when necessary. Technical success was evaluated by en bloc and R0 resection rates, and safety by procedure‐related adverse events. Secondary outcomes included comparison of procedure time between the UEMR and UIEMR groups and descriptive comparisons of baseline characteristics. Exploratory analyses were performed to identify factors associated with conversion from UEMR to UIEMR. En bloc resection was defined as the removal of the lesion in a single piece without macroscopic residual tumor. R0 resection was defined as en bloc resection with histologically negative lateral and vertical margins. Procedure‐related adverse events included intraprocedural perforation, delayed perforation, and delayed bleeding. Intraprocedural perforation was defined as endoscopic visualization of extraluminal tissue or intraperitoneal free air on computed tomography (CT). Delayed perforation was defined as intraperitoneal free air on CT within 14 days after the procedure. Delayed bleeding was defined as hematochezia requiring endoscopic hemostasis within 14 days after the procedure. Emergency colonoscopy was performed for repeated hematochezia, hemoglobin decrease ≥2 g/dL, or hemodynamic instability. Intraprocedural bleeding that was controllable by endoscopic hemostasis was not considered an adverse event. Procedure time was defined as the interval from completion of diagnostic image acquisition to lesion resection and clip closure. In the UIEMR group, the time spent attempting UEMR was included in the total procedure time.

### 2.5 Statistical Analysis

Continuous variables were expressed as median (interquartile range [IQR]) and categorical variables as number (percentage). Comparisons between the UEMR and UIEMR groups were performed using the Mann–Whitney U test for continuous variables and Fisher's exact test for categorical variables. Exploratory analyses were conducted to identify factors associated with conversion from UEMR to UIEMR. Univariable analyses assessed the association between each clinical factor and conversion to UIEMR. Considering the limited number of conversion events and risk of overfitting, a multivariable logistic regression model was constructed using a small number of clinically relevant variables predefined independent of univariable results. The model included three factors: lesion location (right‐sided vs left‐sided colon), tangential view, and morphology (Isp vs non‐Isp). Adjusted odds ratios (ORs) with 95% confidence intervals (CIs) were calculated. All tests were two‐sided, and a *p* < 0.05 was considered statistically significant. Analyses were performed using R version 4.5.1 (R Foundation for Statistical Computing, Vienna, Austria).

## 3 Results

### 3.1 Baseline Data

During the study period, a total of 56 non‐pedunculated colorectal lesions measuring 10–20 mm in 44 patients were analyzed. Resection was completed using UEMR alone in 31 lesions, whereas conversion from UEMR to UIEMR was required in 25 lesions during the procedure. Baseline patient and lesion characteristics are summarized in Table 1. Baseline characteristics of lesions treated with UEMR or converted to UIEMR are shown in Table 2. The median endoscopic lesion size was 14 mm (IQR 11–16 mm). Median pathological size, when measurable, was 12 mm (IQR 10–15 mm).

**TABLE 1:** Baseline characteristics of patients and colorectal lesions.

	Total (*n* = 56)
Male, *n*/*N* (%)	33/44 (75.0)
Age, years, median (IQR)	71 (66–76.5)
Total no. of lesions, *n*	56
Lesion location, *n* (%)	
Right–sided colon	19 (33.9)
Left–sided colon	37 (66.1)
Lesion size, mm, median (IQR)	14 (11–16)
Morphology, *n* (%)	
0‐Is	18 (32.1)
0‐Isp	25 (44.6)
0‐IIa + Is	5 (8.9)
0‐IIa	8 (14.3)
Antithrombotic use, *n* (%)	7 (12.5)
Final resection method, *n* (%)	
UEMR	31 (55.4)
UIEMR	25 (44.6)

Patient–based variables were calculated per patient (*n* = 44), and lesion–based variables per lesion (*n* = 56).

Abbreviations: IQR, interquartile range; UEMR, underwater endoscopic mucosal resection; UIEMR, underwater injection endoscopic mucosal resection.

**TABLE 2:** Baseline characteristics of lesions treated with underwater endoscopic mucosal resection (UEMR) or converted to underwater injection endoscopic mucosal resection (UIEMR).

	UEMR (*n* = 31)	UIEMR (*n* = 25)	*p*‐value
Sex, male, *n* (%)	24 (77.4)	20 (80.0)	1.000
Age, years, median (IQR)	70.0 (63.5–74.5)	72.0 (69.0–79.0)	0.086
Antithrombotic use, *n* (%)	5 (16.1)	2 (8.0)	0.443
Lesion location, *n* (%)			0.004
Right–sided colon	5 (16.1)	14 (56.0)	
Left–sided colon	26 (83.9)	11 (44.0)	
Tangential view, *n* (%)	3 (9.7)	12 (48.0)	0.002
Opposite to the channel side, *n* (%)	2 (6.5)	5 (20.0)	0.223
Inner curvature side, *n* (%)	1 (3.2)	4 (16.0)	0.161
Behind a fold, *n* (%)	2 (6.5)	10 (40.0)	0.003
On a fold, *n* (%)	4 (12.9)	9 (36.0)	0.058
Isp morphology, *n* (%)	20 (64.5)	5 (20.0)	0.001
Lesion size, mm, median (IQR)	14.0 (11.0–16.0)	13.0 (12.0–14.0)	0.473
Lesion size ≥15 mm, *n* (%)	12 (38.7)	5 (20.0)	0.155

Abbreviations: IQR, interquartile range; UEMR, underwater endoscopic mucosal resection; UIEMR, underwater injection endoscopic mucosal resection.

There were no significant differences between the two groups with respect to age, sex, or use of antithrombotic agents. In contrast, several treatment‐related factors differed significantly between the groups. Significant differences were observed in lesion location (right‐sided vs left‐sided colon; UEMR group: 5/26, UIEMR group: 14/11), endoscopic view (tangential vs non‐tangential; UEMR group: 3/28, UIEMR group: 12/13), and macroscopic type (Is/Isp/IIa+Is/IIa; UEMR group: 8/20/1/2, UIEMR group: 10/5/4/6). In multivariable regression analysis, right‐sided colon location (p = 0.005), tangential endoscopic view (p = 0.001), and macroscopic types other than Isp (p = 0.016) were independently associated with conversion to UIEMR during UEMR (Table 3). In univariable analysis, a lesion located behind a fold was significantly associated with conversion to UIEMR. However, this factor was not included in the prespecified multivariable model and therefore was not assessed for independence. No other baseline characteristics differed significantly between the groups.

**TABLE 3:** Exploratory univariable and multivariable logistic regression analyses of predefined factors for conversion from underwater endoscopic mucosal resection (UEMR) to underwater injection endoscopic mucosal resection (UIEMR).

	Univariable		Multivariable	
	OR (95% CI)	*p*‐value	Adjusted OR (95% CI)	*p*‐value
Lesion location (Right‐sided vs. Left‐sided colon)	6.37 (1.67–28.60)	0.004	11.00 (2.26–70.59)	0.005
Tangential view (+ vs. −)	8.27 (1.81–53.44)	0.002	31.62 (5.10–343.28)	0.001
Opposite to the channel side, *n* (%)	3.54 (0.52–40.72)	0.223		
Inner curvature side, *n* (%)	5.55 (0.50–290.10)	0.161		
Behind a fold, *n* (%)	9.27 (1.67–97.68)	0.003		
On a fold, *n* (%)	3.70 (0.86–19.26)	0.058		
Morphology (Isp vs. non‐Isp)	0.14 (0.03–0.53)	0.001	0.12 (0.02–0.59)	0.016
Lesion size, mm, median (IQR)	0.92 (0.76–1.10)	0.473		
Lesion size ≥15 mm, *n* (%)	0.40 (0.09–1.52)	0.155		

Abbreviations: CI, confidence interval; OR, odds ratio; UIEMR, underwater injection endoscopic mucosal resection.

### 3.2 Procedure‐related Outcomes

En bloc and R0 resection were achieved using UEMR alone in 31 lesions. In the remaining 25 lesions, conversion from UEMR to UIEMR enabled successful en bloc and R0 resection. Consequently, the overall en bloc and R0 resection rates were both 100% (56/56). Histopathological diagnoses included low‐grade adenoma, high‐grade adenoma, serrated lesions, and carcinoma (pTis/pT1). Six lesions were diagnosed as carcinoma, all of which were resected en bloc. One lesion was identified as T1b carcinoma and was resected by UIEMR; both horizontal and vertical margins were negative, with a vertical margin distance of 1250 µm. The patient subsequently underwent additional surgical resection, and no residual tumor or lymph node metastasis was observed. The median procedure time was 4.5 (3.0–7.1) min in the UEMR group and 5.6 (4.7–8.0) min in the UIEMR group, with a significantly longer procedure time observed in the UIEMR group (*p* = 0.047). Procedure‐related outcomes, including resection status, procedure time, and histopathological diagnosis, are summarized in Table 4.

**TABLE 4:** Procedure‐related outcomes of the step‐up strategy.

	Total (*n* = 56)	UEMR (*n* = 31)	UIEMR (*n* = 25)	*p*‐value
Resection status (overall), *n* (%)				
En bloc resection	56 (100)	31 (100)	25 (100)	—
R0 resection	56 (100)	31 (100)	25 (100)	—
Procedure time, min, median (IQR)	5.1 (3.8–7.3)	4.5 (3.0–7.1)	5.6 (4.7–8.0)	0.047
Complications, *n* (%)				
Delayed bleeding <48 h	0 (0)	0 (0)	0 (0)	—
Delayed bleeding ≥48 h	0 (0)	0 (0)	0 (0)	—
Intraprocedural perforation	0 (0)	0 (0)	0 (0)	—
Delayed perforation	0 (0)	0 (0)	0 (0)	—
Histology, *n* (%)				0.139
Adenoma, LGD	35 (62.5)	19 (61.3)	16 (64.0)	
Adenoma, HGD	9 (16.1)	6 (19.4)	3 (12.0)	
Serrated lesions	6 (10.7)	1 (3.2)	5 (20.0)	
Carcinoma (pTis/pT1)	6 (10.7)	5 (16.1)	1 (4.0)	

En bloc resection was defined as the removal of the lesion in a single piece without macroscopic residual tumor; R0 resection was defined as en bloc resection with histologically negative lateral and vertical margins.

Procedure time was defined as the interval from completion of diagnostic image acquisition to lesion resection and clip closure; in the UIEMR group, time spent attempting UEMR was included. Abbreviations: LGD, low‐grade dysplasia; HGD, high‐grade dysplasia; UEMR, underwater endoscopic mucosal resection; UIEMR, underwater injection endoscopic mucosal resection.

### 3.3 Adverse Events

No delayed bleeding, intraprocedural perforation, or delayed perforation occurred in either UEMR or UIEMR (Table 4). All cases of intraprocedural bleeding were successfully managed with endoscopic clip placement alone, and no patients required blood transfusion, interventional radiology, or surgical intervention.

## 4 Discussion

UEMR exploits the buoyancy of the mucosa and submucosa in an underwater environment to transform non‐pedunculated or flat lesions into a relatively polypoid configuration, thereby facilitating snare capture. By filling the intestinal lumen with water, luminal distension is reduced while the muscularis propria is thought to maintain a circular configuration, which may lower the risk of perforation. Underwater techniques offer several advantages, including improved maneuverability, reduced discomfort and sedation, enhanced visualization, and attenuation of thermal injury via a heat‐sink effect [15, 16]. Nevertheless, the reported R0 resection rates for intermediate‐sized (10–20 mm) non‐pedunculated colorectal lesions remain variable; a Japanese randomized controlled trial reported a rate of 69%, and meta‐analyses have shown rates of approximately 60% [8, 9].

In the present study, we adopted a step‐up strategy using UEMR first, with conversion to UIEMR when stable snaring or adequate resection margins could not be achieved. This strategy resulted in a high en bloc resection rate, with histologically negative margins reported in all lesions. These findings suggest that timely conversion to UIEMR may improve treatment completeness when adequate margins are difficult to obtain with UEMR alone. With UIEMR, submucosal expansion under underwater conditions facilitates injection, and the subsequent reduction in mucosal tension and improved cushioning allow more stable anchoring of the snare tip. Uniform spread of the injectate within the resection field may stabilize tissue resistance, contributing to more consistent electrosurgical cutting. Although these results appear favorable, they should be interpreted with caution. Pathological evaluation was based on routinely processed specimens without systematic stretching and pinning fixation, which may have affected margin assessment. Therefore, the R0 resection rate may have been overestimated. Furthermore, particularly challenging lesions, such as recurrent lesions or lesions in anatomically difficult locations, were not included in this cohort, which may have also contributed to the favorable technical outcomes observed in this study. Another factor is the relatively large proportion of Isp lesions in this cohort (25/56), which differs from many previous UEMR studies. Underwater luminal collapse may make the flat component of Isp lesions less prominent, facilitating snare capture. Accordingly, the inclusion of these lesions may have partially influenced the overall technical results.

Analysis of treatment‐related factors showed that conversion to UIEMR was more frequently observed in lesions located in the right colon, those observed in a tangential view, and those with morphologies other than Isp. This finding is consistent with previously reported determinants of technical difficulty during colonoscopic polypectomy [17]. These factors may partly reflect overlapping procedural aspects of technical difficulty during UEMR rather than independent determinants. However, multicollinearity among these variables was low (all variance inflation factors <1.5), suggesting that each factor may represent different aspects of procedural difficulty. Under these conditions, stable snaring with UEMR may be difficult; therefore, UIEMR may serve as a useful rescue option.

No delayed bleeding or intraprocedural or delayed perforation occurred in this study. In addition to the heat‐sink effect inherent to the underwater environment, a consistent procedural strategy—cutting current alone, thin‐wire snares, and clip closure after resection—may have contributed to the low incidence of adverse events. However, given the relatively small sample size, the safety of this strategy should be interpreted with caution.

Recently, Hirai et al. reported favorable outcomes of UIEMR when used as a standalone procedure for colorectal lesions, including safety and a high en bloc resection rate, and suggested the potential to secure adequate vertical margins [18]. Consistent with that report, we observed a high en bloc resection rate and favorable safety. Furthermore, in our series, one T1 colorectal cancer was resected using UIEMR with histologically negative lateral and vertical margins; however, the clinical utility of UIEMR for invasive cancer remains uncertain. Hirai et al. evaluated UIEMR primarily as a standalone procedure and did not present explicit criteria for selecting UIEMR, whereas our study evaluated UIEMR as a rescue option within a step‐up strategy. Applying UIEMR uniformly to all cases could increase procedural complexity and prolong procedure time when adequate snaring is achievable with UEMR alone. In the present study, we predefined explicit intra‐procedural criteria for “UEMR difficulty” and evaluated the effectiveness and safety of using UEMR first and selectively applying UIEMR to difficult cases. This approach leverages the advantages of UEMR while providing appropriate rescue in technically challenging situations. Our findings complement and extend the work of Hirai et al. by demonstrating the usefulness of UIEMR as a rescue option for UEMR‐difficult cases. Several techniques have been proposed for UEMR‐difficult cases, including snaring synchronized with peristalsis, stepwise lesion contraction under water [19, 20], and underwater tip‐in EMR [21]. A recently reported technique termed “tip‐fixing UEMR” has also been described for difficult UEMR cases [22]. UIEMR can be positioned as an additional rescue option that complements these techniques.

Procedure time prolongation associated with conversion to UIEMR was limited, suggesting this strategy is a practical option to improve treatment completeness. Nonetheless, excessive submucosal injection during UIEMR may unnecessarily expand the resection field; therefore, injection volume should be kept to the minimum required. In anatomically narrow segments, particularly the sigmoid colon, submucosal injection may reduce the working space, and indications should be considered carefully. Rectal lesions were included in the left‐sided colon category, and the results suggest that this approach may also be applicable to rectal lesions. Lesions involving the dentate line were not included.

This study has several limitations. First, it was a single‐center retrospective study with a limited sample size, and potential selection bias associated with a single operator cannot be excluded. Furthermore, all procedures were performed under standardized conditions; therefore, the favorable outcomes may represent a best‐case scenario, and the generalizability may be limited. Pathological evaluation was based on routinely processed specimens without systematic stretching and pinning fixation; therefore, margin assessment may have been limited, and the R0 resection rate may have been overestimated. The lack of follow‐up data on recurrence is another limitation, and the long‐term effectiveness of this strategy could not be fully evaluated. Application of the predefined criteria for UEMR difficulty may remain partly subjective, potentially affecting reproducibility. Moreover, there was no parallel control group treated with conventional EMR or a predefined UIEMR‐for‐all strategy. Therefore, further prospective studies and external validation are needed to confirm the clinical utility of this step‐up strategy. Finally, the multivariable logistic regression analysis should be interpreted cautiously because the number of conversion events was limited, resulting in wide confidence intervals and potential model instability or overfitting.

## 5 Conclusion

A step‐up strategy using UEMR first with selective conversion to UIEMR for 10–20 mm non‐pedunculated colorectal lesions was feasible and safe. This approach may be useful when stable snaring or clear margin visualization is difficult with UEMR alone. However, without a control group, the comparative effectiveness of this strategy and the optimal treatment approach remain unclear. Prospective comparative studies are needed to clarify its clinical role.

## Author Contributions

**Akihiro Yoshida**: investigation, data curation, formal analysis, and writing – original draft; **Tsuyoshi Ono**: investigation; **Satoshi Ito**: investigation; **Kenta Watanabe**: writing – review and editing; **Toru Okuzono**: investigation; **Katsunori Iijima**: conceptualization, methodology, writing – review and editing, and supervision. All authors approved the final version of the manuscript.

## Funding

The authors have nothing to report.

## Ethics Statement

This study was approved by the institutional review board of our institution (approval number: 3) and was conducted in accordance with the principles of the revised Declaration of Helsinki.

## Conflicts of Interest

The authors declare no conflicts of interest.

## Consent

As this was a retrospective study, the requirement for informed consent was waived.

## Supporting information




**Supporting Video 1**: An underwater endoscopic mucosal resection (UEMR) procedure being performed.

**Supporting Video 2**: An underwater injection endoscopic mucosal resection (UIEMR) procedure being performed.
